# Fluoride concentration data in groundwater resources of Gonabad, Iran

**DOI:** 10.1016/j.dib.2018.09.062

**Published:** 2018-09-27

**Authors:** Abbasali Karimi, Majid Radfard, Morteza Abbasi, Ali Naghizadeh, Hamed Biglari, Vida Alvani, Mokhtar Mahdavi

**Affiliations:** a Health Services Management, School of Public Health, Social Determinants of Health Research Center, Yasuj University of Medical Sciences, Yasuj, Iran; b Research Center for Health Sciences, Institute of Health, Department of Environmental Health, School of Health, Shiraz University of Medical Sciences, Shiraz, Iran; c Behvarz Training Center, Torbat Heydariyeh University of Medical Sciences, Torbat Heydariyeh, Iran; d Medical Toxicology and Drug Abuse Research Center (MTDRC), Birjand University of Medical Sciences (BUMS), Birjand, Iran; e Department of Environmental Health Engineering, School of Public Health, Social Development & Health Promotion Research Center, Gonabad University of Medical Sciences, Gonabad, Iran; f Department of Environmental Engineering, School of Engineering and Technology, Murdoch University, Western Australia, Australia; g Social Determinates of Health Research Center, Saveh University of Medical Sciences, Saveh, Iran

**Keywords:** Groundwater, Fluoride, Fluorosis, Gonabad, Water quality, GIS

## Abstract

The data was obtained from fluoride ion concentrations determined in groundwater sources of Gonabad. A number of 144 samples from 12 drinking groundwater wells located in different regions at the mid of spring, summer, autumn and winter from each regions, were collected from spring 2016 to spring of 2017. The fluoride ions in the samples were determined through the SPADNS colorimetric method at a wavelength of 580 nm. Dispersion of fluoride ions in terms of concentration in groundwater resources of Gonabad was planned using geographic information system (GIS) base on inverse distance weighted (IDW) method. The data showed that lowest and highest concentration of fluoride ions were determined 0.46 in winter and 1.56 mg/l in summer season, respectively. The average, max and min amount of fluoride concentration in groundwater resources of Gonabad were 0.67 ± 0.03, 1.56 and 0.46 mg/L, respectively. The average chemical parameters concentration of Potassium, Sodium, Magnesium, Calcium, Phosphate, Nitrate, Bicarbonate, Sulfate and Chloride were 6.61 ± 1.2, 281.17 ± 3.08, 26.75 ± 2.55, 68.14 ± 3.89, 0.2 ± 0.04, 10 ± 1.83, 275.02 ± 7.20, 282.08 ± 5.04 and 235 ± 5.83 mg/L, respectively.

**Specifications table**

**Table:** 

Subject area	Environmental Sciences
More specific subject area	Groundwater chemistry
Type of data	Table and figure
How data was acquired	The concentration of fluoride ions were determined by the SPADNS colorimetric method. The dispersion of fluoride ions was planned using GIS software v10.3 base on inverse distance weighted (IDW) method.
Data format	Raw, analyzed
Experimental factors	Groundwater samples from 12 different areas of Gonabad were collected from drinking water wells sources, private and government hand pumps in the year 2016–17. All sampling sites were selected with a view to cover the entire area of the study area.
Experimental features	The samples collection and fluoride ions analysis conducted according to the standards methods of water and wastewater treatment analyze handbook.
Data source location	Gonabad, Iran
Data accessibility	Data are included in this article

**Value of the data**

• Managing drinking water quality is crucial for treatment plant operators. The data can be used by drinking water quality researchers [1], [2].
• Accreditation to a recognized flouride quality standard may be essential for dealing with certain customers or complying with legislation. This data can be used to illustrate the dispersion of flouride ions concentration in groundwater resources of Gonabad county [3].
• This data can be used to show how much fluoride ion levels from this drinking water may affect the consumer [4].
• The data can be used to show the fluoride ions concentration at risk level where it is, since it may affect the health of consumers for operators to make better decision in treatment method selection [5].

## 1 Data

**Fig. 1:**
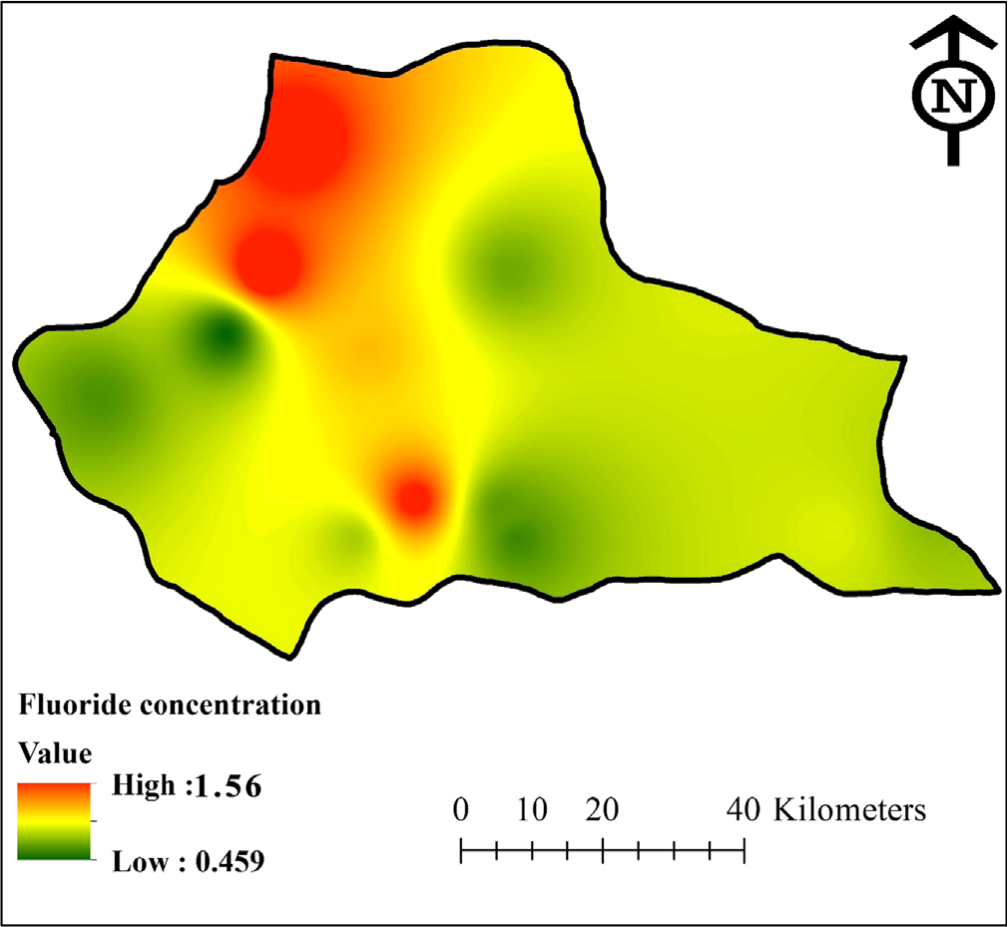
Fluoride ions dispersion in groundwater resources of Gonabad.

**Fig. 2:**
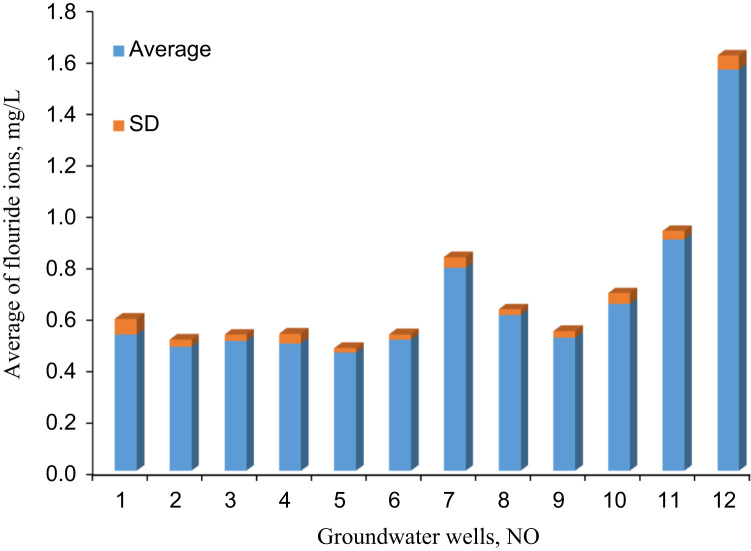
Average of fluoride ions concentration in groundwater resources of Gonabad.

The data in Table 1, Table 2 shows the descriptive statistics of fluoride ions and other chemical parameters concentration in samples. Fig. 1 shows the distribution of fluoride ions in Gonabad groundwater resources. Fig. 2 shows the average along with Standard deviation of fluoride ions concentration in groundwater resources of Gonabad. The fluoride ions concentration in critical regions illustrated with dark red color in Fig. 1 Standards set for the concentration of fluoride in drinking water by various organizations are different. The differences are associated with seasonal changes [6]. Factors affecting the standard concentration of fluoride ions in drinking water have a direct relationship with air temperature and water consumption per capita [7], [8], [9]. In other words, the amount of drinking water intake in the warm months is more than its amount in the cold months [10], [11]. According to the standard of drinking water in Iran, the optimum concentration of fluoride in drinking water is 0.7 mg/L in warm months while it is 2.1 mg/L in the cold months [12]. The average fluoride concentration of groundwater resources in Gonabad falls in the range of 0.66–0.67 mg/L. Table 1 shows the fluoride ions concentration in different groundwater regions. The maximum and minimum concentration of fluoride ions in all regions were 1.62 and 0.44 mg/L, respectively. The average of maximum was 0.7 mg/L, the minimum was 0.64 mg/L and the average fluoride concentration was 0.67 mg/L. The average concentration of fluoride in groundwater resources used for drinking water in Gonabad is higher than the standards of the World Health Organization, and the current standard of United States of America and Europe [13], [14], [15]. In comparison to the standard of drinking water in Iran, the annual average of fluoride ion deficiency is about 0.5 to 1 mg/L [16]. The data also showed that the average, max and min amount of fluoride concentration in groundwater resources of Gonabad were 0.562 ± 0.23, 0.18 and 1.08 mg/L, respectively. The average chemical parameters concentration of Potassium, Sodium, Magnesium, Calcium, Phosphate, Nitrate, Bicarbonate, Sulfate and Chloride were 6.61 ± 1.2, 281.17 ± 3.08, 26.75 ± 2.55, 68.14 ± 3.89, 0.2 ± 0.04, 10 ± 1.83, 275.02 ± 7.20, 282.08 ± 5.04 and 235 ± 5.83 mg/L, respectively.

**Table 1:** The chemical parameters concentration in groundwater resources of Gonabad, mg/L.

**No**	K^+^		Na^+^		Mg^++^		Ca^++^		PO_4_^-3^		NO_3_^-^		HCO_3_^-^		SO_4_^-2^		Cl^-^	
	Ave	STD	Ave	STD	Ave	STD	Ave	STD	Ave	STD	Ave	STD	Ave	STD	Ave	STD	Ave	STD
1	7	1	230	3.23	26.46	3.56	78.4	4.6	0.15	0.02	11	1	324.15	10.34	230	4	220	3.7
2	3	0.5	197	1.78	19.68	2.6	48.8	2.9	0.42	0.04	9	3	304.65	6.22	150	5.2	140	7.45
3	8	2	270	2.54	34.08	5.1	88.8	6.1	0.16	0.05	12	1	333.91	8.45	255	7.1	300	5
4	2	0.7	192	1.34	14.88	1.56	37.6	2.39	0.12	0.09	14	3	246.16	10.2	60	2.9	55	6.4
5	4	1	190	3.1	15.36	3.55	32.8	1.78	0.16	0.04	8	2	233.97	9.13	80	1.5	100	9.3
6	10	1.5	402	5.02	39.36	2.89	104.1	5.5	0.22	0.03	12	1	283.45	4.45	480	2.2	368	2.7
7	4	1.57	420	3.87	24.96	1.3	36.8	2.37	0.16	0.02	5	1	275.43	5.56	360	4.6	350	4
8	9	2.1	342	4.65	41.28	4.02	85.2	2.55	0.14	0.04	8	2	411.89	7.32	320	6	300	5.2
9	5	0.48	240	0.2	25.92	2.4	78.4	8.2	0.17	0.07	10	2	219.35	6.65	270	8.5	230	9.8
10	5	1	309	2.33	18.72	0.32	68.3	1.63	0.25	0.03	8	3	202.52	8.24	380	5.1	245	2.4
11	12	1.5	312	5.32	35.36	1.89	58.4	5.2	0.21	0.03	11	1	262.45	4.25	450	4.2	298	7.7
12	5	2.1	270	3.6	24.96	1.45	100.1	3.4	0.26	0.05	12	2	202.29	5.6	350	9.2	214	6.3
Ave	6.167	1.29	281.17	3.08	26.75	2.55	68.142	3.89	0.2	0.04	10	1.83	275.02	7.201	282.08	5.04	235	5.83
Min	2	0.48	190	0.2	14.88	0.32	32.8	1.63	0.12	0.02	5	1	202.29	4.25	60	1.5	55	2.4
Max	12	2.1	420	5.32	41.28	5.1	104.1	8.2	0.42	0.09	14	3	411.89	10.34	480	9.2	368	9.8

** NO_2_ and CO_3_ in all samples were zero.

**Table 2:** The fluoride concentration in groundwater resources of Gonabad, mg/L.

**NO**	Spring	Summer	Autumn	Winter	**Average**	**SD**	**Min**	**Max**
1	0.60	0.49	0.48	0.58	0.53	0.06	0.48	0.60
2	0.45	0.51	0.47	0.50	0.48	0.03	0.45	0.51
3	0.52	0.48	0.49	0.53	0.51	0.02	0.48	0.53
4	0.52	0.53	0.49	0.44	0.50	0.04	0.44	0.52
5	0.47	0.45	0.48	0.45	0.46	0.02	0.44	0.48
6	0.48	0.50	0.51	0.53	0.51	0.02	0.48	0.53
7	0.75	0.75	0.83	0.81	0.79	0.04	0.75	0.83
8	0.58	0.63	0.60	0.61	0.61	0.02	0.58	0.63
9	0.53	0.48	0.51	0.54	0.52	0.03	0.48	0.54
10	0.60	0.70	0.63	0.65	0.65	0.04	0.60	0.70
11	0.87	0.88	0.92	0.94	0.90	0.03	0.88	0.94
12	1.56	1.62	1.49	1.54	1.56	0.05	1.57	1.62
Min	0.45	0.45	0.47	0.44	0.46	0.02	0.44	0.48
Average	0.66	0.67	0.66	0.68	0.67	0.03	0.64	0.70
Max	1.56	1.62	1.49	1.54	1.56	0.06	1.56	1.62

## 2 Experimental design, materials and methods

Gonabad, with an area of 5902 square kilometres, lies up the north of Birjand and 270 km off the south of Mashhad and it is near Pakistan (Fig. 2). The city has three towns with the names of Kakhk, Bajestan and Bidokht. With consideration to the geographical location of the Gonabad county, it becomes clear that this city has a hot and dry climate and with an average annual rainfall of 65 mm and temperature –14 up to 44.6 °C, the city suffers from water shortages.

Water samples were prepared from different dug wells. Selected locations were quite close to water wells that are used for drinking. All of the sampling, transferring and analyzing methods have been carried out according to the water and wastewater standard methods. The water samples were prepared from 12 drinking water wells out of 18 wells that allowed for sampling and reporting due to security issues. Four samples (three repetitions) were collected at the mid of spring, summer, autumn and winter from each well, and 144 samples were totally taken overall (one year monitoring (from spring 2016 to spring of 2017)). Water samples were taken from the well water pipe in the middle of the day. The water samples were transferred to chemical laboratory of the Gonabad University of medical sciences in a cool box immediately. Sampling was conducted with one‑liter polyethylene bottles which were immersed in nitric acid for 24 h then washed with 10 percent HCL and finally washed with distilled water. It has to be mentioned that before the collection of the samples, sampling containers had been rinsed at least three times with water [16], [17]. In the laboratory the fluoride ions in the samples were measured using the SPADN method at a wavelength of 580 nm by HACH (spectrophotometer DR 5000 Company, USA). The SPADN reaction is based on the degree of red color loss resulting from zirconium SPADN reaction in proportion to the concentration of fluoride ion [18], [19]. The scatter of fluoride concentration were mapped by Geographic Information System software v 10.3 using IDW method for illustrate the critical fluoride concentration level in groundwater area [20].
