# Supplementary material for: Fluoride concentration data in groundwater resources of Gonabad, Iran
Source: Data Brief. 2018 Sep 27;21:105–10. doi: 10.1016/j.dib.2018.09.062 (PMC6186961; doi:10.1016/j.dib.2018.09.062)
Supplement: Supplementary file 1 — Supplementary material [file mmc1.docx]

Dear editor(s)

Data in Brief

None of the authors of this paper have any conflicts of interest to declare.

Sincerely yours

Corresponding author

Hamed Biglari
